# Fear of dying dirty: Intimate care encounters during COVID-19 pandemic in South African context

**DOI:** 10.4102/hsag.v28i0.2317

**Published:** 2023-08-25

**Authors:** Simangele Shakwane

**Affiliations:** 1 Department of Health Studies, Faculty of Humanities, University of South Africa, Pretoria, South Africa

**Keywords:** admission, death and dying, COVID-19 pandemic, culture, religion, intimate care, patient, touch

## Abstract

**Background:**

Physical distancing, personal protective equipment (PPE) and hand hygiene were encouraged during the pandemic of COVID-19. However, personal hygiene procedures for patients admitted to hospitals, such as assisted baths, oral care and elimination, were neglected.

**Aim:**

This study aimed to describe intimate care and touch experiences for patients admitted to the hospital during the COVID-19 pandemic lockdown.

**Setting:**

This study was conducted in the medical and surgical units of two hospitals in Gauteng province.

**Methods:**

A generic qualitative approach was used to explore and describe the patients’ intimate care and touch experiences during the COVID-19 hard lockdown. In-patient individuals above 18 years were purposively sampled. Twelve patients aged between 28 and 60 years participated in semi-structured interviews. Data were analysed using thematic analysis.

**Results:**

Three central themes emerged from the data: (1) Keeping away from the body, (2) Who is touching my body? and (3) Fear of dying dirty - a sense of losing bodily dignity. The participants felt that the nurses were trying to avoid them, as they were seen as potential carriers of the COVID-19 pandemic.

**Conclusion:**

The cleanliness of a patient’s body gives them a sense of self-respect and dignity. Nurses should find ways to ensure that patients receive quality intimate care and touch, even during situations such as the pandemic.

**Contribution:**

Patients’ religious or cultural beliefs and anxieties about dying dirty should be acknowledged and respected in nursing care to provide quality bodily care for all patients.

## Introduction

Intimate care is one of the significant elements of the nursing profession that makes it different from other healthcare professionals, as nurses spend more time with their patients and provide safe, individualised, holistic and comprehensive care (Jordan et al. 2022). Caring is a core attribute of nursing, including physical, mental and emotional care (Shin & Yoo 2022). Much literature has been dedicated to describing the COVID-19 pandemic and its progress, with the main concern being the direct or indirect transmission through contact with infected individuals and contaminated objects (Garba, Lubuma & Tsanou 2020).

Many countries adopted strategies to minimise and control the spread of the COVID-19 pandemic. ‘Social or physical distancing’ aimed to reduce clustering and interactions among individuals and communities (Mbunge 2020; Shah et al. 2021). It is an umbrella term for the limitation and restriction of personal interactions and movement (Feldman et al. 2022), which led to limiting health workers’ interactions with patients and minimising the hospitalisation of patients to prevent respiratory droplet transmission (Jamaludin et al. 2020). Regarding hygiene, the focus was on good hand hygiene practices, as this was considered an important measure of risk reduction practices to prevent the transmission of pathogens in healthcare facilities (Moore et al. 2021). Therefore, nurses wore personal protective equipment (PPE), such as gowns, face masks, goggles, face shields and gloves, while working with patients (Deliktas Demirci, Oruc & Kabukcuoglu 2021). The South African Nursing Council (SANC) provided a directive for the mandatory provision of PPE to protect nurses and patients while rendering nursing care (SANC 2021).

Patients’ hygiene related to bodily cleanliness and excretion (i.e. urination and defaecation) is essential to maintaining the patient’s dignity and integrity. Intimate care is classified as the closeness of a patient and nurse at a physical, psychological and spiritual level where touch cannot be avoided (Shakwane & Mokoboto-Zwane 2020b). Thompson et al. (2021) emphasised that intimate care is person-centred and requires creating and maintaining a relational space that promotes integrity. Intimate care is task-oriented touch to areas of patients’ bodies that might cause them discomfort, anxiety and fear (O’Lynn, Cooper & Blackwell 2017). Nurses need to touch patients to provide bodily care; the body plays a role in assigning meaning to experiences of illness and guides what action needs to be performed (Ohajunwa & Mji 2018). Touch is essential in nursing as it communicates care (Pedrazza et al. 2018) and is a medium to connect with the patient to achieve nursing goals (Mainey et al. 2018). During basic nursing care provision, intimate care conflict can be experienced by both the provider of care and the recipient. The Model of Shakwane and Mokoboto-Zwane (2020b) for facilitating the teaching of intimate care suggests that intimate care conflict exists through diversity such as age, gender, religion and cultural beliefs of a nurse and a patient. As the tasks are hands-on, a patient’s physical and social space is invaded (Thompson et al. 2021). Therefore, a therapeutic nurse-patient relationship should be created through negotiating and establishing trust, respect and dignity during intimate care execution. Understanding patients’ cultural and religious beliefs and social norms is essential when providing culturally acceptable intimate care and touch (Shakwane & Mokoboto-Zwane 2020a).

During hospitalisation, the power rests with the healthcare provider, and touch can be perceived as a manifestation of that power, causing the patients to interpret it negatively (Gleeson & Timmins 2005). During the COVID-19 pandemic lockdown, nurses could not provide basic nursing care that involved touch owing to the changing clinical practice guidelines on isolation, physical distancing and wearing of PPE (Moore et al. 2021). This situation caused exhaustion and discomfort for nurses (Gordon, Magbee & Yoder 2021). A bed bath is a form of therapeutic care that allows for close relationships between the nurse and patients (Avilar et al. 2021). The patients had to put aside their embarrassment and allow their bodies to be exposed to professionals of both sexes, without being given any options to alleviate their discomfort. A lack of patient-centred care led to an absence of intimate care and touch during the pandemic because of nurses’ increased workload and their inability to provide standard nursing care (Bergman et al. 2021). The prevention and control of the COVID-19 pandemic also led to the breach of patients’ confidentiality, privacy and integrity (Sun et al. 2021). During admission, patients experienced a loss of relationship, personal control, identity and privacy (Jordan et al. 2022). Intimate care and hygiene care had to be provided following prescribed guidelines for the pandemic to avoid the risk of contamination and promote patient safety (Avilar et al. 2021).

Patients’ human dignity was infringed, as they were isolated from both their families and the nurses caring for them; so when a patient died, the individual was separated from other humans, without any consideration of their beliefs about death and what happens to them after death. Many individuals believe that certain bodily rituals need to be performed before or shortly after a patient dies (Lobar, Youngblut & Brooten 2006; Qian & Jiang 2022). Unfortunately, patients who died in the hospital during the height of the pandemic were simply placed in a sealed plastic bag, which was never opened by the family members. Consequently, the relatives never got a chance to bid their loved ones farewell or conduct their cleansing rituals before burial. The World Health Organization (WHO) speaks of maintaining the dead person’s dignity, and this should include respecting the cultural and religious traditions of the family (WHO 2020). However, the amendments to the *South African National Health Act 61 of 2003* under the *Disaster Management Act 57 of 2002* added stipulations on the viewing of human remains. According to section 28(1), no person was allowed to contact or touch the human remains; in subsection (2), the families were not allowed to wash or prepare the human remains of their loved ones (Republic of South Africa 2020). In traditional African society, death is attached to sacredness; however, the eruption of COVID-19 pandemic influenced the ritual practices (Okechi 2017). Healthcare providers should create space for ideas on African culture regarding clinical interventions and acknowledge the African cultural knowledge system (Singh, Bhagwan & Bhagwan 2020). Creating this space will assist in their preparedness for future disasters and pandemics.

The literature has focused on nurses’ experiences during COVID-19 (Bergman et al. 2021; Gordon et al. 2021; Shah et al. 2021), and, sadly, the patient’s hygiene and touch-based care were neglected in favour of risk prevention and curing measures. Thus, the aim of this study was to explore and describe the experiences of intimate care and touch during the COVID-19 pandemic lockdown in two selected hospitals in South Africa.

## Research methods and design

### Study design

This study forms part of a larger study on the implementation and evaluation of intimate care facilitation for nursing students in South Africa. Therefore, the author will share the primary qualitative phase 1 on patients’ experiences of admission during the COVID-19 pandemic lockdown, focusing on procedures involving intimate care and bodily touch of patients in medical, surgical and COVID-19 rooms. A qualitative research approach with an explorative, descriptive and contextual design assisted in gaining a better understanding of the personal experiences of those directly involved. The author recognised the value of participants’ unique viewpoints, which could be understood only within the context of their experiences and worldview (Castleberry & Nolen 2018). Thus, the author explored and described the experiences of patients in medical, surgical and COVID-19 rooms about intimate care and touch during the COVID-19 pandemic lockdown in two selected hospitals in South Africa.

### Setting

The study was conducted in two hospitals in Gauteng province. Specific units that were targeted were the surgical and medical units, including the COVID-19 rooms. Only patients who were COVID-19-negative during the study were recruited. The selected hospitals created a special entry site where patients were screened for the COVID-19 virus. The patient was admitted to the COVID-19 high-care rooms if the virus was detected. After 7 days, the test was repeated; if the results were negative, the patients were moved to COVID-19 rooms awaiting admission to the main wards (medical or surgical). These sites were used to relieve congestion in the hospitals and have a progressive system before a patient was admitted to the general wards in the hospitals.

### Study population and sampling strategy

The study population comprised the individuals admitted to medical and surgical units during the COVID-19 pandemic lockdown. Purposive sampling refers to selecting research participants who fulfil the research aims and have knowledge and experience of the phenomenon under study (Doyle et al. 2020). The inclusion criteria were as follows: any patients above 18 years of age who were admitted to medical and surgical units during levels 3–5 of the lockdown between November 2021 and March 2022, admitted for 10 or more days in the nursing unit; in addition, they had to be of sound mind and not on any narcotic (sedative) medication. The unit managers assisted with the identification of patients who met these inclusion criteria. The exclusion criteria were as follows: patients not of legal age, meaning less than 18 years old, who required the approval of a parent or legal guardian in line with the social distancing guidelines, patients who were admitted less than 10 days in the specific unit and were disoriented or on narcotic medication.

### Data collection

Semi-structured interviews outlined the topics and questions, and no rigid rules were enforced (Adhabi & Anozie 2017). The questions were formulated using literature on caring for the body during COVID-19. They were controlled by the study’s main objective, which was to explore and describe the intimate care experiences, especially during the strict pandemic lockdown. The handling of these interviews depended on how the interviewees responded to the researcher’s question or issue. Individual semi-structured interviews were conducted on the verandas of the relevant units to maintain confidentiality and limit patient movement. Data collection was conducted from December 2021 to March 2022 in 10 units; at this time, South Africa was at level 3 of the COVID-19 lockdown. The unit managers assisted the researcher in selecting participants who met the criteria, especially in excluding individuals who were disoriented and on sedative medication. The participants were individually recruited, and the information about the study (purpose and how they will participate) was explained to them. Those who were willing to partake in the study were given informed consent to sign before the commencement of a semi-structured interview. Two data collectors were trained in conducting semi-structured interviews and adhering to institutional COVID-19 preventive measures, such as keeping a physical distance of 1.5 m, wearing face masks and practising hand hygiene using alcohol-based hand sanitisers. The data collectors signed a confidentiality agreement to protect the participants’ identities and dignity. The interviews were restricted to one unit per day to avoid transmission of the virus. Twelve patients aged between 28 and 60 years were admitted for more than 10 days in the relevant units. The four main questions presented in Table 1 were posed to all the participants. Each data collector had a digital recorder. The interviews were digitally audio-recorded. After each interview, the data were transferred to the researcher’s laptop and deleted from the digital audio recorder. The author conducted verbatim transcriptions of all interviews.

**TABLE 1:** Interview guide questions.

Question numbers	Questions
Q1	What has been your experience with the basic care of your body requiring touch during the COVID-19 pandemic?
Q2	What were your challenges relating to the care provided to your body?
Q3	What were your most significant concerns about the physical care of your body?
Q4	What do you think can be done differently in the future, should there be another pandemic?

Probing questions were asked for clarity, and then, a summary of the participant’s responses was given to ensure the correctness of the information collected. Data saturation was reached at eight interviews, and four more interviews were conducted to confirm data saturation. Twelve participants were interviewed: seven were female, four were male, and one was classified as neither male nor female. The duration of each interview was 15–20 min. Data were analysed 24 h after data collection by transcribing all audio-recorded interviews. This allowed for the observation of data saturation, implying that no new information was developed during sampling and analysis (Hamilton 2020). Data were stored in a password-protected zipped file on the researcher’s personal laptop. The raw data were available to an independent coder who signed a confidentiality agreement to keep the research information private.

### Data analysis

An explorative, descriptive and contextual qualitative design was used in the study. Thematic analysis was used to analyse the textual data from semi-structured interviews. The key characteristic of thematic analysis is the systematic coding process, examining the meaning and provision of a description of social reality by creating themes (Vaismoradi et al. 2016). The goal of thematic analysis is to understand the pattern of meaning from data in lived experiences (Sundler et al. 2019). The author and independent coder used thematic analysis to understand the importance of the participants’ lived experiences in the study by doing the following:

- **Achieving familiarity with the data through open-minded reading:** The author and independent coder read the verbatim transcripts to become familiar with the data.
- **Searching for meaning and themes:** The meanings of lived experiences were sought and marked. They were further described by comparing similarities and dissimilarities between the meanings. The meanings were organised into a pattern and merged into themes.
- **Organising the themes into meaningful categories:** The themes explicitly described the meanings of the participants’ lived experiences of intimate care and touch during admission in the COVID-19 lockdown period.

### Trustworthiness

Trustworthiness was ensured by applying *credibility, transferability, dependability* and *confirmability* principles. The application of these trustworthiness principles is discussed in Table 2.

**TABLE 2:** Application of trustworthiness.

Principles	Definitions	Applications
Credibility	Concerns the extent to which the research’s conclusions can be viewed as believable (Sundler et al. 2019).	Relevant participants were recruited using participative sampling based on the researcher’s judgement of the participants’ experiences on intimate care and duration of hospital admission during strict lockdown during the COVID-19 pandemic. An open relationship was created between the participants and data collectors during the recruitment process, and data were collected in the units where the participants were admitted. During data analysis, an independent coder assisted in developing themes and interpreting the findings.
Transferability	Focuses on the usefulness and relevance of the findings from one context to another (Nassaji 2020).	The author presented a thorough and rich description of the research activities, allowing the reader to measure whether the findings are sound and whether the study adds new knowledge. Furthermore, the geographic location of the study, number and characteristics of participants, research methods and time frames for data collection are completely described allowing for its applicability to a new context.
Dependability	In qualitative research, this principle indicates that the study should be reported so that others can arrive at similar interpretations if they review the data (Nassaji 2020).	The author carefully documented the study’s research activities, findings and conclusions for the reader to determine proper research practices were followed and whether future research can be repeated in a different context.
Confirmability	It concerns how others confirm the researcher’s interpretation and conclusion (Nassaji 2020).	In terms of confirmability, the audit trail was used to document all steps taken regarding the research process.

Note: Please see the full reference list of the article, Shakwane, S., 2023, ‘Fear of dying dirty: Intimate care encounters during COVID-19 pandemic in South African context’, *Health SA Gesondheid* 28(0), a2317. https://doi.org/10.4102/hsag.v28i0.2317, for more information.

### Ethical considerations

Ethical clearance was obtained from the College of Human Sciences Research Committee at the University of South Africa (certificate number 90414357_CREC_CHS_2021). Approvals were also received from the Gauteng Department of Health, the health district and the selected hospitals. The two data collectors and an independent coder signed a confidentiality agreement to protect participants’ personal and sensitive information. All procedures performed were per ethical standards: the participants were informed about the purpose of and their involvement in the study (autonomy); written informed consent was then obtained from all participants, and they were informed that the interviews were audio-recorded for research purposes. Personal information, such as the names of participants and institutions, was coded to ensure anonymity and confidentiality. The participants understood that participation was voluntary and could withdraw at any stage. Two participants voluntarily withdrew after the completion of demographic data. One changed the health condition during the interview, and it was aborted to allow the medical personnel to provide the required care.

## Results

### Characteristics of participants

Twelve participants were interviewed: seven were female, four were male, and one was classified as neither male nor female. Their ages ranged from 28 to 60 years, and they were admitted to medical and surgical units for more than 10 days. Table 3 presents a summary of the characteristics of the participants.

**TABLE 3:** Summary of participants’ characteristics.

Characteristics	Values (*n*)	%
**Gender**		
Male	4	33.30
Female	7	58.30
Other	1	8.33
**Age ranges (years)**		
28–35	2	16.60
36–45	4	33.60
46–55	3	25.00
56–60	3	25.00
**Cultural and language preferences**		
Zulu (isiZulu)	5	41.70
Sepedi (Northern Sotho)	2	16.60
Swati (isiSwati)	3	25.00
Ndebelele (isiNdebele)	2	16.60
**Unit where admitted**		
Medical	7	58.30
Surgical	5	41.70
**Number of days hospitalised**		
10–15	7	58.30
16–20	3	25.00
> 21	2	16.60

## Findings

Three main themes emerged from the findings: (1) Keeping away from the body; (2) Who is touching my body? and (3) Fear of dying dirty – a sense of losing bodily dignity. The participants’ codes present the gender and age of the participant: M (Male), F (Female) and O (Other) non-classified gender. For example, M-25 means a 25-year-old male.

### Theme 1: Keeping away from the body

Intimate care requires the nurse and patient to be in close physical proximity, while basic nursing requires touch. The COVID-19 pandemic was classified as an airborne disease transferable through droplets (Jamaludin et al. 2020). Social distancing led to avoid physical contact with other people, especially patients in hospital settings.

In being admitted during the strict COVID-19 pandemic lockdown, the patients experienced physical distancing and felt as if they were being blamed for spreading the virus. They expressed their experiences as follows:

‘Nurses had to keep away from us [*patients*]; they spoke of maintaining a social distancing there was a measure to prevent the spread of COVID-19 …’ (P2, Male, 25 years old)

‘There was fear to touch us or even closer to us as patients. [*Nursing*] care was based on nurses protecting themselves from contracting COVID 19 from us [*patients*]; we were seen as carriers of the virus.’ (P1, Female, 28 years old)

The healthcare practitioners were covered with personal protective clothing, so the patients could not identify or hear the nurses:

‘Everyone was covered all over their bodies; it was difficult to identify who was a nurse or doctor. It is very much important that when you offer your services, you still need to introduce yourself to the patient because nurses did not even have their name tags on because of the gowns, masks and everything they were wearing.’ (P5, Male, 40 years old)

‘It was very difficult to hear or even read the lips of the person giving care. I did not even hear the instructions simply because a mask covers a person, a face shield or goggles. So I find it very, very, very difficult.’ (P2, Female, 33 years old)

When needing to provide physical care, the nurses did so hastily; the important thing was eliminating the spread of the COVID-19 pandemic and minimising the infection. However, the basic care that required bodily cleanliness was neglected:

‘[*Q*]uick care was given – medication was given preference – things like bathing or cleaning of patients took a back seat, they [*nurses*] were afraid to come to physical contact with us. When I was first admitted, I was isolated and alone. Social interaction between us [*nurses and patients*] was not done as the nurses had to consider everyone having COVID-19. They were providing care fearfully.’ (P3, Male, 50 years old)

Social or physical distancing was prescribed to minimise the spread of the pandemic. Nurses had to wear protective clothing for self-protection; they were frontline soldiers in a viral war and had to protect themselves. The PPE created a barrier to basic nursing care, such as bathing and elimination.

### Theme 2: Who is touching my body?

The provision of intimate care requires nurses to touch the patients’ bodies. Therefore, the patient should be informed about the care that will be provided to their bodies. During intimate care, gender preference is essential for the patient to make an informed decision about their touched bodies.

Wearing PPE made it difficult for the patients to see and know who was providing care. As established earlier, touch cannot be avoided when providing intimate care. Knowing the person touching them was crucial for the patients to decide on what they would allow to be done to their bodies. The participants did not know their caregivers:

‘I have been long in hospital, they [*nurses*] all protected themselves, I don’t know who was doing what with my body. I did not see or hear – I just saw white ghosts moving …’ (P13, Female, 57 years old)

‘When I arrived, I was in the COVID-19 isolation ward; you just saw white movements; they did whatever they needed to do with your body and then exited as fast as possible and spent as little time. There was not enough communication to form a healthy relationship. I felt more alone and scared as I did not know or see the person caring for me.’ (P1, Other, 30 years old)

Patients’ gender preferences were ignored during intimate care as the nurses wore PPE, making it difficult for the patients to know whether the nurse was male or female:

‘When you get into the unit, …, everyone is in white or blue gowns. So you don’t know which one is a male or female. I was also wearing a mask. It was hard for me because I was in the surgical ward, admitted for rape. So I was scared as I did not know whether the person walking around was my rapist coming to finish me off [*kill me*]. Since they kept checking my private parts, I could see the big hands in the gloves that it was a man. I wish I knew the people touching my most private parts.’ (P4, Female, 36 years old)

Patients admitted to the hospital need to know who is touching them and be able to make informed choices about who touches them and how their bodies are touched. Physical distancing and wearing PPE should not diminish the bodily dignity of the patient.

### Theme 3: Fear of dying dirty – A sense of losing bodily dignity

Cleanliness gives a person a sense of dignity. Moreover, intimate care and touch allow patients to feel comfortable about their bodies. During the strict lockdown of COVID-19 pandemic, the patients’ bodily care was neglected as nurses were trying to protect themselves from contracting the COVID-19 pandemic, while the patients who could not fulfil their physical cleanliness needs did not receive sufficient care. The principle of treating each person as COVID-19 positive was followed. As a result, the patients were scared to die in an unclean bodily state:

‘I was not afraid to die, but I was afraid to die dirty; the passage to my ancestors would have been dark.’ (P4, Male, 60 years old)

‘My family could not come to help me with a full body bath as I was only given a disposable wet towel to wipe my face and private parts. I felt dirty. I prayed to be better so that I could have a bath. A few days ago, I could get out of bed, and now I can slowly walk to the bathroom and wash in the small basin. I feel a bit clean and can look at myself again.’ (P7, Female, 48 years old)

The proverb ‘Cleanliness is close to godliness’ was relevant to bodily cleanliness in the case of the participants. Cleansing the body after earthly life is essential in African cultures. One participant provided insight into the importance of washing the body before burial:

‘[*M*]any rituals are performed on the body. The final cleansing of the body is conducted in the funeral home by a delegated elderly, male or female, depending on the gender of the dead person. The person’s body is covered with their favourite clothing …’ (P6, Female, 52 years old)

When a person dies outside the home, the body and spirit need to be reconnected to have a safe passage to their ancestors:

‘When a person dies in the hospital or away from home, before the burial of the body, the elders must fetch the spirit of the person to be joined with the body. If the body is buried alone, the spirit will wonder and will not be an ancestor …’ (P5, Male, 58 years old)

The participants’ value of a dignified death involved having a clean body and physical and spiritual connectedness. However, for many patients who died in the hospital, their bodies were sealed in a bag to contain the spread of the virus. Thus, African cultures and values were compromised in saving lives.

## Discussion

This study presents the participants’ experiences of intimate care and touch during the COVID-19 pandemic lockdown. The cleanliness of the human body offers a person a sense of self-respect and dignity. However, the participants experienced isolation and loneliness as the nurses tried to keep themselves safe from contracting the virus. Social distancing meant that the nurses had to keep away from the patients’ bodies by avoiding close contact and touch. Unfortunately, this action compromised the primary care provided to vulnerable patients. This was in line with the WHO’s (2020) recommendation for social distancing between individuals to prevent respiratory droplet transmission during human interaction (Jamaludin et al. 2020; Shah et al. 2021). In Bergman et al. (2021), registered nurses expressed the challenges of ensuring physical distancing as they could not provide the best standard of care. The participants acknowledged the importance of PPE to prevent the virus’ spread, but such equipment concealed the caregiver’s identity and prevented communication with the patient. In terms of section 28 of the *Disaster Management Act* (Republic of South Africa 2020), no person was allowed to contact or touch the human body of a patient without wearing the appropriate PPE. The South African Nursing Council (2021) provided a directive for the mandatory provision of PPE for frontline workers. Clearly, nurses should not have risked their lives by working without the appropriate PPE during the COVID-19 pandemic. However, physical distancing led to incomplete care (Deliktas Demirci et al. 2021), creating fear within the nursing profession, as most were infected with the virus, and some even died (Akkuş et al. 2022).

The question, ‘Who is touching my body?’ calls for reflection on the importance of identifying the person providing nursing care and showing respect for the patient’s body. When nurses wore PPE, such as gloves, gowns and goggles, it restricted their communication and interaction with patients; thus, there was less nursing engagement (Deliktas Demirci et al. 2021). The provision of intimate care involves hands-on work, which infringes on a patient’s personal and social space (Thompson et al. 2021). Patients had to submit to embarrassing situations during intimate care procedures, such as bed baths, by exposing their bodies to professionals without being able to make choices that could alleviate their discomfort (Avilar et al. 2021). Furthermore, the participants were not informed about how and where they would be touched. Arcadi et al. (2021) attested that the meaning of care changed during the pandemic; the physical distancing diminished the care that consisted of closeness, communication and patient protection. The studies of Gordon et al. (2021) and Wittenberg et al. (2021) confirmed that using PPE challenged the clinical care of patients and decreased patient-centred care. When providing care requiring touch, the patients ought to be treated with respect by the nurses, thereby acknowledging their value as a person (Bridges et al. 2021) and the meaning they attach to their bodies. Most nursing care requires the patient’s body to be touched; therefore, a nurse must inform the patient of what procedure they need to perform and ask for the patient’s permission to uphold the ethical principle of autonomy. This allows the patient to make an informed choice and exercise control over their body (Dowdell & Speck 2022). Open and effective communication about touch is necessary to avoid misinterpretation (Kelly et al. 2018) and assist the patient in feeling physically and emotionally safe (Kokokyi, Klest & Anstey 2021).

The COVID-19 pandemic lockdown led to nurses’ neglecting intimate care procedures that promote patient cleanliness and respect for the human body. The participants’ greatest fear ‘was not to die, but to die dirty’ in the hospital. The WHO recommendation of social distancing and personal hygiene focused on cleaning hands with soap and water and limiting healthcare professional engagements with patients to decrease the spread of the COVID-19 pandemic (Jamaludin et al. 2020). The cultural and religious values in African societies define the position and role of individuals, thus presenting challenges to gender equality. The African human personhood is influenced in its formation and development by cultural and socialisation processes within local traditions and customs (Nwoye 2017). A person means that the self has meaning when it is in a creative relationship with others and the rest of creation (Klaasen 2017). In Africa, becoming an ancestor after death is a desire for individuals and can be achieved through living a meaningful life (Ekore & Lanre-Abass 2016). This includes having an acceptable or good death, which allows for social adjustments and personal preparation (Gire 2014). Ancestors are the living-dead compassionate spirits who are blood-related to people who believe in them; they act as mediators between the living and the dead (Mokgobi 2014). Avoiding engaging in things that provoke their ancestors’ anger is important; therefore, people control themselves by fear and anticipatory consequences of acting contrary to the prescriptions sanctioned by their ancestors and spiritual agencies (Nwoye 2017). Dying clean for the 60-year participant refutes the idea that the response to one’s death is fear (Gire 2014) and affirms that death is associated with the sacredness of life after death. The focus on curing and the pharmaceutical treatment of COVID-19 left nurses unprepared for intimate care and touch while promoting and advocating for patient safety. Social distancing and hygiene must be redefined in terms of the unprecedented COVID-19 pandemic.

Various rituals are performed on the body after death to assist in the smooth passage to a new life as an ancestor; this includes closing the eyes and washing the body in preparation for burial (Martin et al. 2013). Death indicates the physical separation of the individual from other humans. The funeral and ceremonies focus on this permanent separation and avoid causing undue offence to the dead (Ekore & Lanre-Abass 2016). Death changes the identity of the person (Gire 2014). The study indicates that the family must wash the body for the last time after a person dies. The WHO (2020) provided critical considerations for the safety and well-being of those who tend to dead bodies. It emphasised the dignity of the deceased’s body, including cultural and religious traditions. It asserted that the families of the deceased should be respected and protected throughout the process (WHO 2020). The prohibition of the family from washing or preparing the human violated the bodily dignity of the deceased. The South African Constitution of 1996 provided hope and fundamental rights for all its people. In South Africa, culture is regarded as a way of acting, thinking and doing things unique to a particular group (Mubangizi 2012). Protection of cultural rights is stipulated in sections 30 and 31 of the Constitution. In terms of section 30, cultural rights are protected to provide individuals with cultural and language rights. In contrast, section 31 refers to a collective dimension, emphasising belonging to a group and community. Therefore, a person does not exist in isolation; each person grows up in a specific social and cultural setting, which influences the individual’s behaviour in the world (Kpanake 2018). The COVID-19 pandemic has limited these rights in terms of a general clause in section 36, which permits limitations that are ‘reasonable and justifiable in an open and democratic society, based on human dignity, equality and freedom’. Therefore, the inability to prepare the body adequately for burial and to maintain the body of the deceased in a dignified manner has been a challenging experience for many. African bereavement rituals render death a broadly dialectical process of shepherding the deceased’s soul through a series of embodied practices performed and involving the bodies of the deceased and the bereaved; the rituals steward the deceased’s soul to an ancestral spiritual realm (Martin et al. 2013). For the majority of African people, conducting proper rituals on the dead body eases the passage to the next life. In this study, the greatest desire of the participants’ relatives was to advocate for bodily hygiene for the participants who were awaiting their turn to be released from their earthly journey. Therefore, the healthcare system must recommit to upholding social, cultural and just practices that respect each person’s humanity and dignity.

### Strengths and limitations of the study

This study has shared the nurses’ and patients’ struggles in navigating intimate care and touch challenges during the COVID-19 pandemic. The desire for the cleanliness of the body symbolises respecting the dignity and integrity of the person. Understanding what was neglected in the quest to save lives allows for learning, reflecting and recreating a safe space for intimate care and touch.

The limitations of the study were the overnight changes imposed in terms of the data collection process; owing to the emergencies, the units did not allow non-health professionals access to the wards, units and patients. Also, patients’ health conditions changed either before or during the interview.

### Recommendations

More research is recommended to understand the effects of physical distancing by healthcare professionals providing intimate care and touch and views of dying dirty during the COVID-19 pandemic. The concept of physical distancing needs to be analysed so that ways can be found to provide high-quality nursing care that delivers intimate care and touch to patients in need. Moreover, nursing professionals advocate for personal hygiene for vulnerable patients and maintain the ethical principles of autonomy, informed consent and patient privacy.

## Conclusion

Being isolated from their family undoubtedly caused enormous stress for the hospitalised patients during the lockdown. Using defensive care to avoid contracting and spreading COVID-19 left patients feeling unwanted, as they were seen as the source of the virus, with the result that the nurses tried their best to avoid touching them. It goes without saying that nurses were faced with ethical dilemmas created by the COVID-19 preventive measures, such as physical distancing and wearing PPE, which led to communication difficulties and possibly even misunderstandings. As the profession prepares for future outbreaks, nurses must be ready to deal with and respect the patient’s physical, cultural and religious needs to maintain patient dignity.

## References

[CIT0001] Adhabi, E.A.R. & Anozie, C.B.L., 2017, ‘Literature review for the type of interview in qualitative research’, *International Journal of Education* 9(3), 86. 10.5296/ije.v9i3.11483

[CIT0002] Akkuş, Y., Karacan, Y., Güney, R. & Kurt, B., 2022, ‘Experiences of nurses working with COVID-19 patients: A qualitative study’, *Journal of Clinical Nursing* 31(9–10), 1243–1257. 10.1111/jocn.15979 34309116 PMC8446967

[CIT0003] Arcadi, P., Simonetti, V., Ambrosca, R., Cicolini, G., Simeone, S., Pucciarelli, G. et al., 2021, ‘Nursing during the COVID-19 outbreak: A phenomenological study’, *Journal of Nursing Management* 29(5), 1111–1119. 10.1111/jonm.13249 33421209 PMC8014333

[CIT0004] Avilar, C.T., Andrade, Í.M.A., Nascimento, C.S., Viana, L.V.M., Amaral, T.L.M. & Prado, P.R., 2021, ‘Nursing care for bed bath in patients with COVID-19: An integrative review’, *Revista Brasileira de Enfermagem* 75(Suppl 1), e20200704. 10.1590/0034-7167-2020-0704 34816965

[CIT0005] Bergman, L., Falk, A.C., Wolf, A. & Larsson, I.M., 2021, ‘Registered nurses’ experiences of working in the intensive care unit during the COVID-19 pandemic’, *Nursing in Critical Care* 26(6), 467–475. 10.1111/nicc.12649 33973304 PMC8242789

[CIT0006] Bridges, C., Duenas, D.M., Lewis, H., Anderson, K., Opel, D.J., Wilfond, B.S. et al., 2021, ‘Patient perspectives on how to demonstrate respect: Implications for clinicians and healthcare organisations’, *PLoS One* 16(4 April), 1–14. 10.1371/journal.pone.0250999 PMC8084197 33914815

[CIT0007] Castleberry, A. & Nolen, A., 2018, ‘Thematic analysis of qualitative research data: Is it as easy as it sounds?’, *Currents in Pharmacy Teaching and Learning* 10(6), 807–815. 10.1016/j.cptl.2018.03.019 30025784

[CIT0008] Deliktas Demirci, A., Oruc, M. & Kabukcuoglu, K., 2021, ‘It was difficult, but our struggle to touch lives gave us strength: The experience of nurses working on COVID-19 wards’, *Journal of Clinical Nursing* 30(5–6), 732–741. 10.1111/jocn.15602 33325080

[CIT0009] Dowdell, E.B. & Speck, P.M., 2022, ‘CE: Trauma-informed care in nursing practice’, *American Journal of Nursing* 122(4), 30–38. 10.1097/01.NAJ.0000827328.25341.1f 35348516

[CIT0010] Doyle, L., McCabe, C., Keogh, B., Brady, A. & McCann, M., 2020, ‘An overview of the qualitative descriptive design within nursing research’, *Journal of Research in Nursing* 25(5), 443–455. 10.1177/1744987119880234 34394658 PMC7932381

[CIT0011] Ekore, R.I. & Lanre-Abass, B., 2016, ‘African cultural concept of death and the idea of advance care directives’, *Indian Journal of Palliative Care* 22(4), 369–372. 10.4103/0973-1075.191741 27803556 PMC5072226

[CIT0012] Feldman, I., Natsheh, A., Nesher, G. & Breuer, G.S., 2022, ‘Social distancing and bacteraemia in the time of COVID-19’, *Internal Medicine Journal* 52(2), 223–227. 10.1111/imj.15560 34617387 PMC8652679

[CIT0013] Garba, S.M., Lubuma, J.M.S. & Tsanou, B., 2020, ‘Modeling the transmission dynamics of the COVID-19 Pandemic in South Africa’, *Mathematical Biosciences* 328(August), 108441. 10.1016/j.mbs.2020.108441 32763338 PMC7402282

[CIT0014] Gire, J., 2014, ‘How death imitates life: Cultural influences on conceptions of death and dying’, *Online Readings in Psychology and Culture* 6(2), 1–22. 10.9707/2307-0919.1120

[CIT0015] Gleeson, M. & Timmins, F., 2005, ‘A review of the use and clinical effectiveness of touch as a nursing intervention’, *Clinical Effectiveness in Nursing* 9(1–2), 69–77. 10.1016/j.cein.2004.12.002

[CIT0016] Gordon, J.M., Magbee, T. & Yoder, L.H., 2021, ‘The experiences of critical care nurses caring for patients with COVID-19 during the 2020 pandemic: A qualitative study’, *Applied Nursing Research* 59(January), 151418. 10.1016/j.apnr.2021.151418 33947512 PMC7946535

[CIT0017] Hamilton, J.B., 2020, ‘Rigor in qualitative methods: An evaluation of strategies among underrepresented rural communities’, *Qualitative Health Research* 30(2), 196–204. 10.1177/1049732319860267 31274057

[CIT0018] Jamaludin, S., Azmir, N.A., Mohamad Ayob, A.F. & Zainal, N., 2020, ‘COVID-19 exit strategy: Transitioning towards a new normal’, *Annals of Medicine and Surgery* 59(October), 165–170. 10.1016/j.amsu.2020.09.046 33024558 PMC7529589

[CIT0019] Jordan, P., Iwu-Jaja, C., Mokoka, E., Kearns, I., Oamen, B., De Lange, S. et al., 2022, ‘Development of a training programme for professional nurses in South Africa – An educational response to the COVID-19 pandemic’, *Nursing Open* 10(1), 377–384. 10.1002/nop2.1273 35713655 PMC9349748

[CIT0020] Kelly, M.A., Nixon, L., McClurg, C., Scherpbier, A., King, N. & Dornan, T., 2018, ‘Experience of touch in health care: A meta-ethnography across the health care professions’, *Qualitative Health Research* 28(2), 200–212. 10.1177/1049732317707726 29235944

[CIT0021] Klaasen, J., 2017, ‘The role of personhood in development: An African perspective of development in South Africa’, *Missionanlia* 45(1), 29–44. 10.7832/45-1-154

[CIT0022] Kokokyi, S., Klest, B. & Anstey, H., 2021, ‘A patient-oriented research approach to assessing patients’ and primary care physicians’ opinions on trauma-informed care’, *PLoS One* 16(7 July), 1–21. 10.1371/journal.pone.0254266 PMC8270182 34242358

[CIT0023] Kpanake, L., 2018, ‘Cultural concepts of the person and mental health in Africa’, *Transcultural Psychiatry* 55(2), 198–218. 10.1177/1363461517749435 29400136

[CIT0024] Lobar, S.L., Youngblut, J.A.M. & Brooten, D., 2006, ‘Cross-cultural beliefs, ceremonies, and rituals surrounding death of a loved one’, *Pediatric Nursing* 32(1), 44–50. 16572538

[CIT0025] Mainey, L., Dwyer, T., Reid-Searl, K. & Bassett, J., 2018, ‘High-level realism in simulation: A catalyst for providing intimate care’, *Clinical Simulation in Nursing* 17, 47–57. 10.1016/j.ecns.2017.12.001

[CIT0026] Martin, J., van Wijk, C., Hans-Arendse, C. & Makhaba, L., 2013, ‘“Missing in action”: The significance of bodies in African bereavement rituals’, *Psychology in Society* 44(1), 42–63, viewed 03 May 2023, from http://www.scielo.org.za/scielo.php?script=sci_arttext&pid=S1015-60462013000100003&lng=en&tlng=en.

[CIT0027] Mbunge, E., 2020, ‘Effects of COVID-19 in South African health system and society: An explanatory study’, *Diabetes and Metabolic Syndrome: Clinical Research and Reviews* 14(6), 1809–1814. 10.1016/j.dsx.2020.09.016 PMC7485444 32956925

[CIT0028] Mokgobi, M.G., 2014, ‘Understanding traditional African healing’, *African Journal for Physical Health Education, Recreation, and Dance* 20(2), 24–34. 26594664 PMC4651463

[CIT0029] Moore, L.D., Robbins, G., Quinn, J. & Arbogast, J.W., 2021, ‘The impact of COVID-19 pandemic on hand hygiene performance in hospitals’, *American Journal of Infection Control* 49(1), 30–33. 10.1016/j.ajic.2020.08.021 32818577 PMC7434409

[CIT0030] Mubangizi, J.C., 2012, ‘A South African perspective on the clash between culture and human rights, with particular reference to gender-related cultural practices and traditions’, *Journal of International Women’s Studies* 13(3), 33–48.

[CIT0031] Nassaji, H., 2020, ‘Good qualitative research’, *Language Teaching Research* 24(4), 427–431. 10.1177/1362168820941288

[CIT0032] Nwoye, N., 2017, ‘An Afrocentric theory of human personhood’, *Psychology in Society* 24, 54–66. 10.17159/2309-8708/2017/n54a4

[CIT0033] Ohajunwa, C. & Mji, G., 2018, ‘The African indigenous lens of understanding spirituality: Reflection on key emerging concepts from a reviewed literature’, *Journal of Religion and Health* 57(6), 2523–2537. 10.1007/s10943-018-0652-9 29909518

[CIT0034] Okechi, O.S., 2017, ‘Culture, perception/belief about death and their implication to the awareness and control of the socio-economic, environmental and health factors surrounding lower life expectancy in Nigeria’, *Acta Psychopathologica* 03(05), 1–10. 10.4172/2469-6676.100128

[CIT0035] O’Lynn, C., Cooper, A. & Blackwell, L., 2017, ‘Perceptions, experiences and preferences of patients receiving a clinician’s touch during intimate care and procedures: A qualitative systematic review’, *JBI Database of Systematic Reviews and Implementation Reports* 15(11), 2707–2722. 10.11124/JBISRIR-2017-003375 29135751

[CIT0036] Pedrazza, M., Berlanda, S., Trifiletti, E. & Minuzzo, M., 2018, ‘Variables of individual difference and the experience of touch in nursing’, *Western Journal of Nursing Research* 40(11), 1614–1637. 10.1177/0193945917705621 28459179

[CIT0037] Qian, M. & Jiang, J., 2022, ‘COVID-19 and social distancing’, *Journal of Public Health (Germany)* 30(1), 259–261. 10.1007/s10389-020-01321-z PMC7247774 32837835

[CIT0038] Republic of South Africa, 2020, ‘Disaster Management Act (57/2002): Regulations made in terms of Section 27(2) by the Minister of Cooperative Governance and Traditional Affairs’, *Government Gazette* 480(43258), 3–36, viewed 12 January 2023, from https://www.gov.za/sites/default/files/gcis_document/202003/4314825-3cogta.pdf.

[CIT0039] South African Nursing Council (SANC), 2021, *Directive for consistent provision of personal protective equipment for frontline nursing staff, Circular 2/2021*, viewed 05 January 2023, from https://www.sanc.co.za/wp-content/uploads/2021/05/Circular-2of2021-Directive-for-consistent-provision-of-PPEs.pdf.

[CIT0040] Shah, S.U., Loo, E.X.L., Chua, C.E., Kew, G., Demutska, A., Quek, S. et al., 2021, ‘Association between well-being and compliance with COVID-19 preventive measures by healthcare professionals: A cross-sectional study’, *PLoS One* 16(6 June), 1–16. 10.1371/journal.pone.0252835 PMC8183980 34097719

[CIT0041] Shakwane, S. & Mokoboto-Zwane, S., 2020a, ‘Demystifying sexual connotations: A model for facilitating the teaching of intimate care to nursing students in South Africa’, *African Journal of Health Professions Education* 12(3), 103. 10.7196/AJHPE.2020.v12i3.1367

[CIT0042] Shakwane, S. & Mokoboto-Zwane, S., 2020b, ‘Promoting intimate care facilitation in nursing education institutions in South Africa’, *International Journal of Africa Nursing Sciences* 13(July), 100226. 10.1016/j.ijans.2020.100226

[CIT0043] Shin, S. & Yoo, H.J., 2022, ‘Frontline nurses’ caring experiences in COVID-19 units: A qualitative study’, *Journal of Nursing Management* 30(5), 1087–1095. 10.1111/jonm.13607 35338532 PMC9115182

[CIT0044] Singh, C., Bhagwan, R. & Bhagwan, P.R., 2020, ‘African spirituality: Unearthing beliefs and practices’, *Social Work / Maatskaplike Werk* 56(4), 0–2. 10.15270/56-4-882

[CIT0045] Sun, N., Wei, L., Wang, H., Wang, X., Gao, M., Hu, X. et al., 2021, ‘Qualitative study of the psychological experience of COVID-19 patients during hospitalisation’, *Journal of Affective Disorders* 278(24), 15–22. 10.1016/j.jad.2020.08.040 32949869 PMC7444461

[CIT0046] Sundler, A.J., Lindberg, E., Nilsson, C. & Palmér, L., 2019, ‘Qualitative thematic analysis based on descriptive phenomenology’, *Nursing Open* 6(3), 733–739. 10.1002/nop2.275 31367394 PMC6650661

[CIT0047] Thompson, G.N., McClement, S.E., Peters, S., Hack, T.F., Chochinov, H. & Funk, L., 2021, ‘More than just a task: Intimate care delivery in the nursing home’, *International Journal of Qualitative Studies on Health and Well-Being* 16(1), 1943123. 10.1080/17482631.2021.1943123 34180776 PMC8245091

[CIT0048] Vaismoradi, M., Jones, J., Turunen, H. & Snelgrove, S., 2016, ‘Theme development in qualitative content analysis and thematic analysis’, *Journal of Nursing Education and Practice* 6(5), 100–110. 10.5430/jnep.v6n5p100

[CIT0049] Wittenberg, E., Goldsmith, J.V., Chen, C., Prince-Paul, M. & Johnson, R.R., 2021, ‘Opportunities to improve COVID-19 provider communication resources: A systematic review’, *Patient Education and Counseling* 104(3), 438–451. 10.1016/j.pec.2020.12.031 33455825 PMC7831717

[CIT0050] World Health Organization (WHO), 2020, ‘Infection Prevention and Control for the safe management of a dead body in the context of COVID-19’, *Journal of Hospital Infection* 104(3), 246–251.32035997

